# Next-generation immune cell therapies for lung cancer: advances in CAR-T, NK, and TIL strategies

**DOI:** 10.3389/fmed.2026.1787086

**Published:** 2026-03-04

**Authors:** Anoud Khan, Amina Mujahid, Aryan Tareen, Ahrar Amin, Saqib Raza Khan, Danial Khan Hadi, Afsheen Raza

**Affiliations:** 1 Department of Medicine, Ziauddin Medical College, Karachi, Pakistan; 2 College of Medicine, Mohammed Bin Rashid University of Medicine and Health Sciences, Dubai Health, Dubai, United Arab Emirates; 3 Division of Medical Oncology, Department of Oncology, Schulich School of Medicine and Dentistry, Western University, London, ON, Canada; 4 Verspeeten Family Cancer Centre, London Health Sciences Centre, London, ON, Canada; 5 Department of Biomedical Sciences, College of Health Sciences, Abu Dhabi University, Abu Dhabi, United Arab Emirates; 6 Cancer Research Institute, Office of Research and Sponsored Programs, Abu Dhabi University, Abu Dhabi, United Arab Emirates

**Keywords:** chimeric antigen receptor natural killer cells, chimeric antigen receptor T cells, lung cancer, next generation immune cell therapies, tumor infiltrating lymphocytes

## Abstract

Lung cancer remains the leading cause of cancer-related mortality worldwide, and current therapies offer limited survival benefit for patients with advanced disease. While immune checkpoint inhibitors and targeted therapies have improved outcomes in some populations, most patients either fail to respond or develop acquired resistance, highlighting the need for more potent and durable immunotherapeutic strategies. Next-generation immune cell therapies, including chimeric antigen receptor T cells (CAR T), chimeric antigen receptor natural killer cells (CAR NK), and tumor-infiltrating lymphocytes (TIL), provide a promising approach for lung cancers that are refractory to conventional treatment. These therapies leverage the patient’s own immune system or engineered immune cells to directly recognize and eliminate malignant cells, while potentially overcoming immunosuppressive tumor microenvironments. This review provides a comprehensive synthesis of recent advances in the design, engineering, and clinical development of CAR T, CAR NK, and TIL therapies in both non-small cell and small cell lung cancer. We discuss preclinical and early clinical studies demonstrating feasibility, safety, and mechanisms of action, including antigen targeting, immune cell persistence, trafficking, and intratumoral activity. Key challenges, such as tumor antigen heterogeneity, immune suppression, limited durability, and off-tumor toxicity, are critically evaluated. We also examine emerging strategies to enhance efficacy, including multi-antigen targeting, armored and logic-gated constructs, regional delivery, combination with checkpoint inhibition or other modulators, and scalable off-the-shelf manufacturing platforms. Collectively, these next-generation immune cell therapies represent a rapidly evolving and translationally relevant approach that may expand therapeutic options, improve survival, and provide durable antitumor responses in patients with lung cancer who have exhausted conventional therapies.

## Introduction

Lung cancer is the leading cause of cancer-related deaths globally, and by 2030, it is projected to cause over 2 million deaths. Recognizing its high mortality has produced immense pressure on the medical world to create treatments that have a better therapeutic response while maintaining a tolerable safety profile (1). According to the World Health Organization (WHO), lung cancer can be classified into two categories based on its cell type: non-small cell lung cancer (NSCLC), which accounts for 80–95% of cases, and small cell lung cancer (SCLC), which accounts for the remaining cases. NSCLC is further categorized into adenocarcinoma, squamous cell carcinoma, and large cell carcinoma (2). Currently, the primary treatment options for lung cancer include surgery, radiation therapy, chemotherapy, immunotherapy, and targeted drug therapy. Yet, the prognosis of lung cancer remains poor, with a 5 year survival of 10–20% (3)

Part of the high mortality is due to late diagnosis, as early disease is typically asymptomatic, and when symptoms do appear, they are often mistaken for benign respiratory conditions. Furthermore, screening uptake remains limited worldwide, as current guidelines recommend low-dose CT (LDCT) scans only for high-risk individuals aged 50–80 years with a 20-pack-year or more smoking history who either currently smoke or quit within the past 15 years (4). Evidence demonstrates that low-dose CT scans significantly reduce lung cancer mortality in this high-risk population; however, a substantial proportion of screening results are false positives, often leading to additional investigations and invasive procedures. Concerns regarding overdiagnosis, patient anxiety, and cumulative radiation exposure have further limited enthusiasm for this screening modality (5).

In addition, definitive local treatments such as surgery or localized radiation may fail due to the aggressive biological nature of lung cancer and its tendency for early metastasis. Even tumors that appear resectable may pose significant challenges because of the lung’s proximity to vital structures (6). Systemic therapies, including chemotherapy and targeted treatments, are further limited by the high molecular heterogeneity of lung tumors, and even when initially effective, tumors frequently acquire new mutations and develop therapeutic resistance (7).

Decades of research spent on understanding cancer have established a key pathway for cancer development: when the body’s immune system fails to recognize and eradicate malignant cells. This failure allows for the uncontrolled proliferation of cancer cells. Recognizing this has encouraged investigators to design strategies that aid the immune system’s natural capacity to detect and destroy such cells. This has led to the development of multiple forms of cancer immunotherapy, most notably immune checkpoint inhibitors, which have revolutionized the treatment of non-small cell lung cancer. Antibodies targeting Programmed Cell Death Protein 1 (PD-1), Programmed death-ligand 1 (PD-L1), and Cytotoxic T-lymphocyte-associated protein 4 (CTLA-4) have produced durable responses in a subset of patients; however, most individuals either fail to respond or develop acquired resistance, highlighting the need for more potent and personalized immune-based strategies (8, 9).

Beyond immune checkpoint blockade, a new class of therapies has emerged based on the direct infusion of tumor-reactive immune cells. Adoptive cellular immunotherapies (ACTs), which include Chimeric Antigen Receptor T-cell therapy (CAR-T), Chimeric Antigen Receptor Natural Killer cell therapy (CAR-NK), and Tumor-Infiltrating Lymphocyte therapy (TIL), can modify a patient’s immune system to target the tumors more effectively (10). These approaches represent a new hope in the treatment of cancers that are resistant to conventional therapies. CAR-T therapy involves modifying a patient’s own T cells by inserting a gene that enables them to express a Chimeric Antigen Receptor (CAR), which then allows the modified T cells to recognize antigens expressed on the surface of cancer cells (10). Since its first clinical approval in 2017, CAR-T therapy has been revolutionary in the treatment of hematologic malignancies, demonstrating high remission rates in B-cell acute lymphoblastic leukemia (ALL) and non-Hodgkin lymphoma (NHL) (11). However, despite its transformative success in B-cell cancers, the application of CAR-T therapy to solid tumors, including lung cancer, remains in its early stages (10). Additionally, there is increasing interest in CAR-NK cell therapies due to their intrinsic cytotoxicity, lower risk of graft-versus-host disease, and potential for off-the-shelf allogeneic use. Likewise, tumor-infiltrating lymphocyte (TIL) therapy is another promising ACT approach that involves harvesting lymphocytes directly from the tumor site, expanding them *ex vivo*, and reinfusing them into the patient (11).

With adoptive cell immunotherapy’s potential to selectively target only cancer cells, the debilitating side effects of chemotherapy and radiation therapy could potentially be reduced. Therefore, this review provides a comprehensive synthesis of the latest advances in next-generation adoptive cellular immunotherapies, critically evaluates emerging clinical evidence in lung cancer, and identifies the key scientific and translational challenges that must be overcome to realize the full therapeutic potential of these approaches in broader clinical practice.

## Methods

This review article analyzes recent literature on the advancement of next-generation immune cell therapies for lung cancer, with a focus on CAR-T, NK, and TIL strategies. Relevant literature published between January 2015 and September 2025 was selected for inclusion. A total of 120 studies were initially reviewed using the databases PubMed, Scopus, and Google Scholar. Key search terms included “CAR-T,” “CAR-NK,” “tumor-infiltrating lymphocytes,” “TIL,” “immune cell therapy,” and “lung cancer.” These terms were combined with Boolean operators to refine the search and capture relevant studies. Boolean operators such as AND, OR, and NOT were used to optimize search results. Synonyms, alternative spellings, and Medical Subject Headings (MeSH) terms were also considered. Only studies published in English were included to ensure consistent data interpretation. Ultimately, 81 studies underwent full-text review, comprising peer-reviewed preclinical and clinical studies, review articles, and meta-analyses investigating CAR-T, NK, or TIL cell-based treatments for lung cancer. Additional sources were identified through citation tracking and manual review of reference lists from relevant publications. After removing duplicates, 74 studies were included in this review article. Data from these sources were qualitatively synthesized and thematically organized by therapy type to illustrate recent scientific progress and future perspectives. The limitations of individual studies were also analyzed and reported to provide a balanced assessment of next-generation immune cell therapies targeting lung cancer.

## Next generation immune cell therapies in cancer treatment

Adoptive Cell Therapy (ACT) has proven to transform oncology in the last decade, allowing patients to use their own or donor-derived immune cells to achieve potent antigen-specific tumor eradication. CAR-T cell therapy, one of the key forms of ACT, has revolutionized the treatment of hematologic malignancies. However, its use in solid tumors, specifically non-small cell lung cancer (NSCLC), remains a critical challenge. There are several primary reasons for their lack of success. A limited antigen pool is one of the core problems. Unlike hematological malignancies, in which well-characterized antigens serve as effective targets, solid tumors rarely express truly tumor-specific antigens. Another key challenge is tumor heterogeneity. Solid tumors consist of multiple subclonal populations, each with a unique genetic profile. These subclonal differences allow for immune evasion, as certain regions of the tumor may lack the targeted antigen. Off-tumor toxicity has played a role in the therapy’s limited success. Solid tumors may express target antigens that are also present in healthy tissues, increasing the risk of on-target, off-tumor effects and resulting in damage to normal organs. Lastly, the tumor microenvironment (TME) encompasses multiple factors that inactivate infiltrating CAR-T cells, including regulatory T cells, myeloid-derived suppressor cells (MDSCs), fibroblasts, and tumor-associated macrophages. The presence of cancer-associated fibroblasts and myeloid cells further forms a physical barrier to infiltrative immune cells through the overexpression of extracellular matrix proteins (12, 13).

However, multiple antigens present in NSCLC have emerged as potential targets for CAR-T therapy, suggesting a promising role for this approach in future lung cancer treatment. Epidermal Growth Factor Receptor (EGFR), which is frequently amplified in various cancer types, has been shown to be a viable target for CAR-T cells (14). Similarly, prostate stem cell antigen (PSCA) and mucin 1 (MUC1), which are often overexpressed in lung cancer, have also been explored as CAR-T targets. Dual targeting of PSCA and MUC1 has demonstrated a reduction in lung tumor burden in preclinical rat models (15). In addition, novel antigens such as erythropoietin-producing hepatocellular carcinoma A2 (EphA2), which is overexpressed in approximately 90% of NSCLC samples and is associated with poorer prognosis, are being investigated as potential CAR-T targets in early-phase clinical trials (16). Furthermore, the integration of molecular imaging techniques to visualize and quantify CAR-T cell trafficking, tumor localization, and *in vivo* persistence is becoming increasingly important for understanding therapeutic mechanisms and optimizing CAR-T cell design in NSCLC (17).

Studies have also focused on developing NK and CAR-NK cell therapies. NK cells are considered safer than traditional CAR-T cells and can be produced on a large scale as “off-the-shelf” treatments (18). The better safety profile of these treatments compared to CAR-T therapy is largely due to the reduced risk of cytokine release syndrome (CRS) and the minimal risk of graft-versus-host disease. This is due to differences in their mechanisms. CRS, which presents with fever, hypotension, and organ dysfunction after CAR-T therapy, is driven by the release of a different set of cytokines; CAR-T cells induce Interleukin-6 (IL-6) release, which drives CRS, whereas NK cells do not. Furthermore, the minimal risk of graft-versus-host disease is because NK cells do not require an antigen-specific T-cell receptor (TCR), unlike T cells. (19). Furthermore, engineering NK cells with CAR constructs adapted to NK-specific signaling domains (DAP10, DAP12, and CD3ζ) augments tumor recognition. DAP10 and DAP12 are intracellular adaptor proteins that activate NK receptors, and the inclusion of such proteins allows for enhanced signal transduction and cytokine production upon antigen recognition. (19). One of the significant logistical challenges of autologous CAR-T therapy is now being addressed through the standardization of NK cells derived from induced pluripotent stem cells (iPSCs) and cord blood (20).

TIL therapy has been one of the earliest form of ACTs, introduced and now being revisited for the treatment of solid tumors (21). This therapeutic strategy entails isolating tumor-infiltrating T cells from the patient, expanding them extensively *ex vivo*, and subsequently reinfusing them into the same individual. By comprising a broad repertoire of T-cell clones, TIL therapy is well suited to overcoming the intrinsic heterogeneity of solid tumors. Importantly, there have been no documented cases of cytokine release syndrome (CRS) associated with TIL therapy to date. In addition, TIL products are enriched for memory T cells that express chemokine receptors such as C-X3-C motif chemokine receptor 1 (CX3CR1) and C-C chemokine receptor type 4 (CCR4), which facilitate effective trafficking to and penetration of solid tumor sites (21). Collectively, these features allow TIL therapy to mount a broad, durable antitumor immune response while maintaining a favorable safety profile compared with other ACT modalities. Recent progress in TIL products for melanoma has sparked new clinical trials evaluating their safety and efficacy in NSCLC, as durable responses have been observed in subsets of patients with high baseline immune infiltration (22).

These immunotherapies show early promise in expanding therapeutic options for lung cancer, although their clinical efficacy remains under active investigation. Current research is focused on improving the antigen specificity, improving persistence, and infiltration within the TME. As this side of ACTs develops, it has become apparent that these next-generation immune cell therapies may offer long-lasting responses and improve survival rates for patients with lung cancer (23).

## Advances in CAR-T cell therapy in lung cancer

**FIGURE 1:**
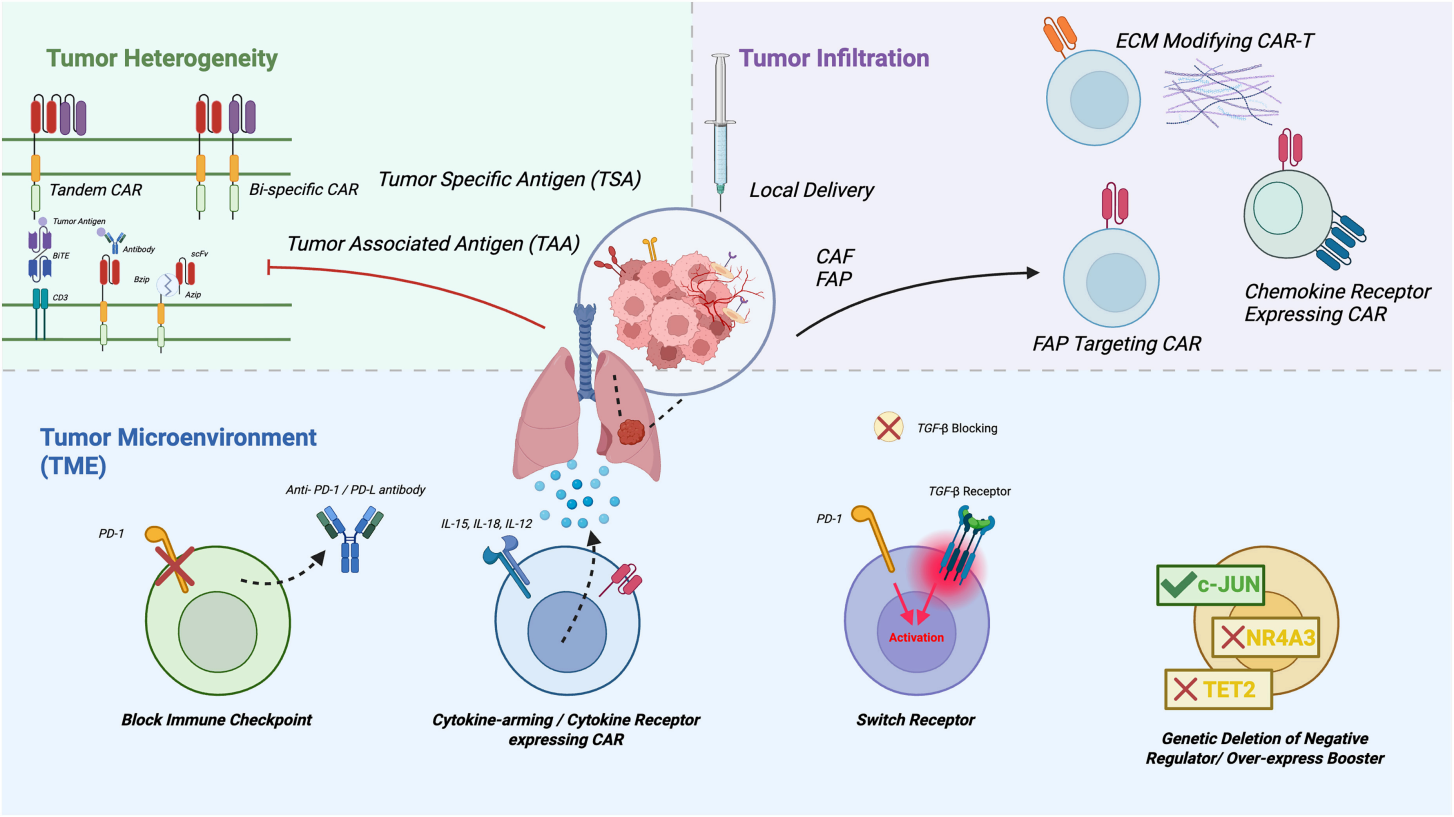
Challenges of CAR-T therapy in solid tumors and engineering strategies to overcome them. CAR-T therapy for solid tumors is hindered by tumor heterogeneity, poor cell infiltration, and an immunosuppressive TME. Multi-antigen targeting strategies, such as tandem and bispecific CARs or BiTE-secreting CAR-T cells, help reduce antigen escape driven by heterogeneous TSA and TAA expression. Tumor penetration can be improved through local delivery, chemokine receptor engineering, FAP-targeting CARs, and ECM-modifying approaches. To counter inhibitory signals within the TME, CAR-T cells can be enhanced by checkpoint blockade, cytokine-arming, switch receptor designs, and genetic removal of intrinsic negative regulators.

As discussed in the overview section, chimeric antigen receptor (CAR) T-cell therapy has advanced from an experimental method to a well-established treatment for hematologic malignancies. It is now a central focus in translational research for lung cancer. Despite its success in leukemia and lymphoma, extending CAR-T therapy to solid tumors such as non-small cell lung cancer (NSCLC) and small cell lung cancer (SCLC) remains challenging due to factors like antigen heterogeneity, a dense and immunosuppressive tumor microenvironment (TME), and limited T-cell infiltration into pulmonary tissues. Consistent with these biological constraints, early clinical studies of CAR-T cell therapy in lung cancer have demonstrated limited objective response rates and short-lived disease control, despite generally manageable toxicity profiles (24). Consequently, advances in CAR-T design, delivery, and manufacturing have been made to overcome these unique obstacles (Figure 1).

A key determinant of CAR-T efficacy in lung cancer is the selection and tuning of antigens. Targets such as carcinoembryonic antigen (CEA), human epidermal growth factor receptor 2 (HER2), epidermal growth factor receptor (EGFR), mesothelin (MSLN), disialoganglioside (GD2), receptor tyrosine kinase-like orphan receptor 1 (ROR1), mucin 1 (MUC1), glypican-3 (GPC3), delta-like ligand 3 (DLL3), and PD-L1 have been explored due to their tumor-associated expression and restricted normal-tissue distribution (25). These antigens have been prioritized based on preferential overexpression in lung tumors and their ability to mediate CAR-dependent T-cell activation in preclinical and early translational studies (25). However, heterogeneous antigen expression within tumors and overlap with normal lung tissues continue to limit therapeutic windows and contribute to off-tumor toxicity.

To mitigate on-target, off-tumor toxicity, a critical concern in lung tissue, recent efforts have employed affinity-tuned CAR constructs and dual-antigen or logic-gated CAR designs that require concurrent recognition of multiple tumor-associated epitopes for activation. Affinity-tuned mesothelin CARs have demonstrated improved tumor selectivity with reduced recognition of normal mesothelial cells, while ligand-based EGFR CARs preferentially target tumor-associated EGFR variants. The translational relevance of these safety-oriented engineering strategies is supported by early mesothelin-directed CAR-T trials, in which restricting antigen sensitivity and systemic exposure was associated with reduced off-tumor pulmonary toxicity (26–28). These approaches aim to expand the therapeutic window for CAR-T therapy in the lung.

Engineering innovations have yielded several next-generation CAR-T platforms designed to overcome immune suppression within the lung tumor microenvironment. Armored CAR-T cells engineered to secrete immunomodulatory cytokines such as IL-12 or IL-18 are intended to enhance T-cell persistence and remodel the TME. Importantly, the majority of evidence supporting cytokine-armored CAR-T cells in lung cancer remains preclinical, with IL-18–secreting DLL3-targeted CAR-T cells demonstrating enhanced persistence and antitumor activity primarily in xenograft models of SCLC (29, 30). These findings provide mechanistic support for cytokine-secreting CAR designs, while highlighting the need for further clinical validation.

Similarly, CAR-T cells co-expressing chemokine receptors such as C-X-C motif chemokine receptor 5 (CXCR5) or C-C chemokine receptor type 6 (CCR6) have been developed to improve tumor homing and infiltration. Preclinical studies in HER2-positive NSCLC models demonstrated enhanced CAR-T trafficking and antitumor activity following chemokine receptor co-expression. Although these modifications significantly improve tumor homing in preclinical settings, clinical trials to date continue to demonstrate limited intratumoral CAR-T persistence in lung cancer, indicating that trafficking enhancement alone may be insufficient to overcome immune exclusion within the pulmonary TME (31). These data suggest that combinatorial strategies will be required to sustain CAR-T activity in solid lung tumors.

Advances in universal or adaptor CAR platforms have further expanded the flexibility and safety of CAR-T therapy in solid tumors. These modular systems allow dynamic control of antigen engagement and CAR-T activity through externally administered adaptors. Such adaptable CAR platforms are particularly attractive for lung cancer, where antigen heterogeneity and dynamic antigen loss have been implicated as contributors to therapeutic resistance in early clinical experience (25, 32). The ability to retarget CAR-T cells without re-engineering may therefore address a major limitation of current lung cancer CAR-T approaches.

Delivery techniques have also evolved to accommodate the anatomical and physiological restrictions of lung cancer. Preclinical and early clinical investigations have demonstrated that regional delivery routes, such as intrapleural or intraparenchymal infusion, increase local control while reducing systemic toxicity. This kind of localized administration shows great promise for isolated thoracic disease and pleural metastases. Clinical trials are being conducted to determine the feasibility of intratumoral CAR-T delivery for MSLN-positive lung cancers, which could provide an alternative when systemic toxicity is a concern (33, 34).

**FIGURE 2:**
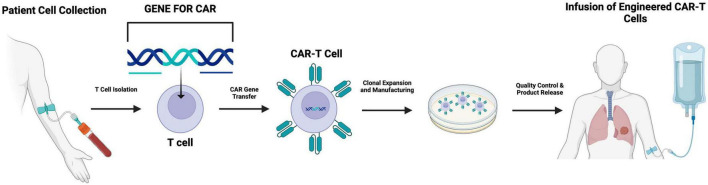
Schematic overview of the CAR-T cell manufacturing and reinfusion workflow in advanced immune cell therapy. The schematic illustrates the primary steps involved in producing CAR-T cell therapy. First, T cells are collected from the patient. These cells are then genetically modified to express a chimeric antigen receptor (CAR). This receptor will enable the immune cell to recognize the tumor cells more effectively. After the engineering, these CAT-T cells are then expanded *ex vivo*, and their potency, purity, and safety are evaluated. Once the cell meets the criteria, the final CAR-T product is infused back into the patient.

From a manufacturing and translational standpoint, progress in allogeneic CAR-T platforms, where endogenous T-cell receptors and HLA are eliminated using gene editing, have cleared the way for off-the-counter treatments that can be rapidly available for patients with advanced lung cancer (35). Despite ongoing issues with persistence and host immunological rejection, these systems promise increased accessibility and shorter manufacturing times. Simultaneously, safety mechanisms such as inducible caspase-9 suicide switches, truncated EGFR markers, and small-molecule gating systems have been implemented to reduce cytokine release syndrome (CRS) and neurotoxicity, which are especially concerning in patients with impaired pulmonary reserve (32) (Figure 2).

Clinical trials using CAR-T cell therapy for lung cancer are still in their early stages, but evidence is growing. Early-phase investigations with MSLN-directed CAR-T cells showed feasibility and controllable toxicity, whereas ligand-based EGFR CAR-T cells showed promising anticancer potential in NSCLC models and preliminary human testing. The IL-18-armored DLL3 CAR has emerged as a promising option for SCLC, with improved persistence and cytotoxicity compared to conventional designs (24). Ongoing logic-gated MSLN studies, such as the AsaeedB694 (EVEREST-2) trial, represent the next wave of translation, as they use built-in inhibitory modules to limit off-tumor activation in HLA-A02-deficient tumor settings (36). Collectively, these studies show consistent progression from early investigative findings to cautious human trials. Table 1 summarizes early-phase clinical trials evaluating CAR-T cell therapies in lung cancer, including targets, patient populations, delivery methods, and key outcomes.

**TABLE 1:** Early-phase clinical trials evaluating CAR-T cell therapies in lung cancer.

Trial (NCT/identifier)	Target/antigen	Phase	Patient population	Delivery/dosing	Key outcomes (safety and efficacy)	References
NCT02414269	Mesothelin (MSLN)	Phase I	Malignant pleural disease, including NSCLC	Intrapleural infusion ± pembrolizumab; dose-escalation	Well tolerated; no DLT in normal organs CAR T detectable > 100 days in 39% Median OS 23.9 months; 1-year OS 83% Stable disease in several patients; 2 complete metabolic responses Pulmonary toxicity in high-dose IV cohorts	(28)
NCT03525782	MUC1 ( ± PD-1 knockout)	Phase I/II	Advanced NSCLC	IV or intratumoral; dose-escalation	Well tolerated; no grade 3–5 toxicities 11/20 patients had stable disease; 9/20 had progressive disease Limited efficacy	(41)
NCT03392064	DLL3	Phase I	Relapsed/refractory SCLC	IV infusion; dose-escalation	Partial response in 1 patient; SD in 2; PD in 1 Favorable safety profile: low-grade CRS, fever, chills, dyspnea CAR-T persistence observed, but limited durability	(43)
NCT03182816	EGFR	Phase I	Advanced NSCLC	IV infusion; piggyBac-transduced CAR-T	3/9 patients achieved PR; 6 had SD Median OS 15.5 months; median PFS 7.1 months. CAR-T persistence detected in peripheral blood	(42)
NCT02706392	ROR1	Phase I	Advanced NSCLC/SCLC	IV infusion	1/8 patients had PR after the s infusion; most had PD by 6 months Generally safe; 1 fatal respiratory failure in extensive disease	(44)

NSCLC, non-small cell lung cancer; SCLC, small-cell lung cancer; CRS, cytokine-release syndrome; DLT, dose-limiting toxicity; OS, overall survival; PFS, progression-free survival; SD, stable disease; PD, progressive disease; PR, partial response.

Emerging objectives of research include combining CAR-T therapy with other immunomodulatory techniques, such as immune checkpoint inhibitors, oncolytic viruses, and radiation, to transform immunologically “cold” lung tumors into inflamed, T-cell-permissive microenvironments (37–39). Furthermore, biomarker-driven patient selection (based on antigen density, TME characteristics, and immune infiltration) and standardized toxicity management are critical for successful clinical translation. According to recent studies, the combination of focused antigen discovery, enhanced cell engineering, optimal transport, and scalable manufacturing is gradually transforming CAR-T therapy into a viable therapeutic option for biomarker-defined subgroups of lung cancer (40). A systematic analysis of CAR T-cell therapy in lung cancer shows that while treatments are generally safe, clinical efficacy remains limited, with few partial responses and most patients experiencing disease progression within months, underscoring the need for optimized CAR designs, targets, and delivery strategies (31). Early-phase clinical experience with CAR-T cell therapy in lung cancer provides important translational insights into how antigen choice, delivery route, and cellular persistence shape clinical outcomes in solid tumors. The phase I trial of regional mesothelin (MSLN)–targeted CAR-T cells delivered intrapleurally in malignant pleural disease (NCT02414269) demonstrated that localized administration, particularly in combination with PD-1 blockade, can support sustained CAR-T expansion and immune activation within the tumor compartment, as evidenced by prolonged CAR-T persistence ( > 100 days in 39% of patients), durable metabolic responses, and favorable survival outcomes, while limiting systemic exposure and off-tumor toxicity observed with intravenous dosing (28). These findings suggest that spatial restriction of CAR-T activity may partially overcome pulmonary toxicity and trafficking barriers inherent to lung cancer. In contrast, systemically delivered CAR-T therapies targeting MUC1 or EGFR in advanced NSCLC (NCT03525782 and NCT03182816) demonstrated that even when safety is acceptable—including the absence of grade 3–5 toxicities and successful peripheral CAR-T engraftment—clinical benefit remains modest, with responses largely limited to stable disease or transient partial responses, highlighting insufficient persistence and functional activity within the immunosuppressive tumor microenvironment (41, 42). In small cell lung cancer, DLL3-directed CAR-T therapy (AMG 119; NCT03392064) provided early pharmacologic evidence of *in vivo* CAR-T expansion and target engagement with manageable cytokine release syndrome, yet responses were short-lived, underscoring the difficulty of achieving durable disease control in a rapidly progressive, antigen-heterogeneous setting (43). Similarly, first-in-human targeting of the oncofetal antigen ROR1 in advanced NSCLC and SCLC (NCT02706392) resulted in limited and non-durable responses despite acceptable safety in most patients, reinforcing the concept that antigen expression alone is insufficient without strategies to enhance CAR-T persistence and resistance to tumor-mediated suppression (44).

Collectively, the outcomes of early-phase CAR-T cell trials in lung cancer reflect disease-specific biological and clinical constraints rather than intrinsic failure of the CAR-T platform itself. The predominance of stable disease, limited durability of responses, and short CAR-T persistence observed across NSCLC and SCLC studies can be attributed to heterogeneous antigen expression, physical barriers to T-cell infiltration, and a profoundly immunosuppressive pulmonary tumor microenvironment characterized by hypoxia, myeloid-derived suppressor cells, and inhibitory cytokine signaling (24, 25, 37). In addition, lung cancer patients often present with compromised respiratory function, which constrains dose escalation and limits tolerance to systemic inflammation, thereby influencing both delivery strategies and safety thresholds in clinical trial design (32, 35). These lung-specific factors help explain why regional delivery, affinity tuning, armored CAR constructs, and combination strategies have emerged as central themes in ongoing translational efforts and underscore the need for disease-adapted CAR-T platforms tailored to the unique biology of lung malignancies (28, 29, 33).

## Advances in NK cell therapy in lung cancer

Natural Killer (NK) cells are a crucial component of the innate immune system, functioning through two primary mechanisms: direct cytolytic activity and immunomodulatory signaling that influences T cells and antigen-presenting cells (APCs). On flow cytometry, NK cells are identified as CD3^–^CD56^+^ and can be further subdivided into two subsets: cytotoxic NK cells (CD56*^dim^*CD16^+^) and cytokine-producing NK cells (CD56*^bright^*CD16^–^) (45).

**FIGURE 3:**
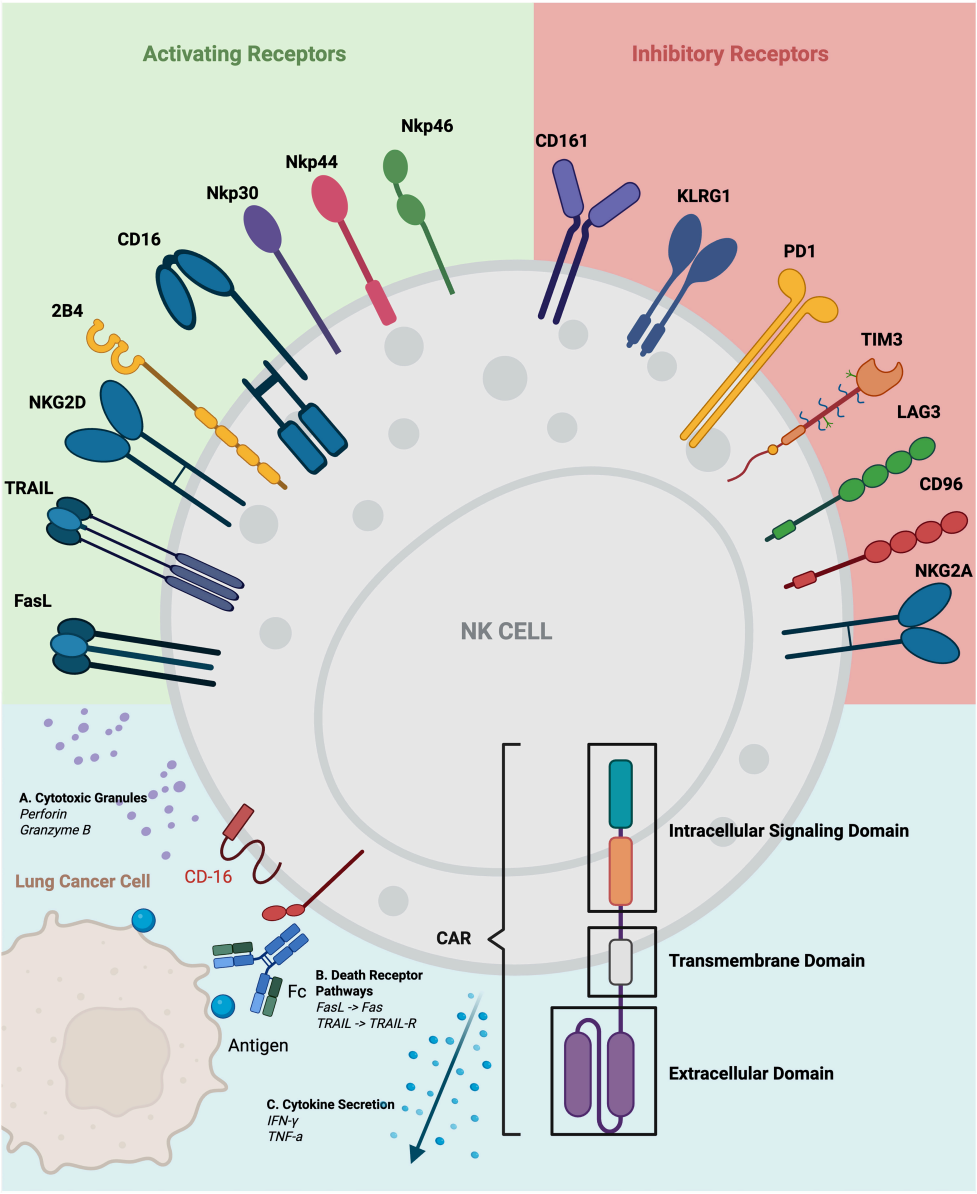
Natural killer (NK) cell receptors and mechanisms of anti-lung cancer activity. This figure depicts the principal activating and inhibitory receptors that govern NK cell effector function. Activating receptors, including NKG2D, CD16, NKp30, NKp44, NKp46, 2B4, TRAIL, and FasL, mediate tumor recognition and initiate cytotoxic granule release, death receptor signaling, and pro-inflammatory cytokine secretion. In contrast, inhibitory receptors such as NKG2A, CD96, LAG3, TIM3, PD-1, KLRG1, and CD161 restrain NK cell activation within the lung tumor microenvironment. A schematic of CAR architecture is included to illustrate how receptor engineering can enhance NK cell specificity and overcome inhibitory signaling to improve antitumor responses.

The cytotoxic activity of NK cells is tightly regulated by a balance between activating and inhibitory signals derived from ligands expressed on target cells and cytokines in the surrounding microenvironment. For example, Major Histocompatibility Complex Class I (MHC-I), or Human Leukocyte Antigen (HLA) in humans, which is abundantly expressed on healthy tissues and provides an inhibitory signal that prevents NK cell activation. Conversely, many cancer cells downregulate MHC-I expression, thereby removing this inhibitory checkpoint and triggering NK cell-mediated cytotoxicity (46) (Figure 3)

Studies have shown a strong association between NK cell activity and cancer incidence. Imai et al. conducted an 11-year-long prospective study that showed a higher baseline cytotoxic activity of NK cells in the blood was associated with significantly lower risk of developing cancer. The study claimed NK cells have a protective role against tumorigenesis (47).

Despite its cytotoxicity toward cancer cells, several mechanisms within the tumor microenvironment suppress the function of NK cells. For example, Transforming Growth Factor-beta (TGF-β) has strong immunosuppressive effects on the NK cells, preventing their activation. In mice with experimental deletion of TGF-β II (TGF-βRII), NK cells can survive and proliferate within the tumor (48). Additionally, the presence of adenosine in the tumor also negatively affects NK cell expansion (35), while acidic conditions can induce NK cell apoptosis (49). Tumors may also escape NK cell-mediated killing by downregulating NKG2D ligands, which are key for NK cell recognition and activation (50).

Regarding lung cancer, NK cell dysfunction is further exacerbated by external and treatment-related factors. Cigarette smoke exposure, which is the leading risk factor for the development of lung cancer, has also been shown to disturb the NK cell cytotoxicity (51). Even chemotherapy, a crucial component of lung cancer treatment, can decrease NK cell activity due to its immunosuppressive effects (52).

Despite the challenges, developments in NK cell therapy focus on reversing tumor-driven immune suppression. As NK-cell therapy advances toward clinical translation, pre-clinical and early clinical evidence continues to expand across diverse approaches (Table 2)

**TABLE 2:** Representative pre-clinical and clinical studies evaluating NK-cell–based immunotherapeutic strategies in lung cancer.

Author (year)	Study type/model	NK-cell approach/intervention	Tumor context/cancer type	Main findings/results	References
Hsu et al. (54) (JCI)	*In vivo* murine tumor models (MHC-I–deficient RMA-S lymphoma and B16F10 melanoma); *in vitro* NK–tumor coculture assays	PD-1/PD-L1 immune checkpoint blockade using monoclonal antibodies	MHC-I–low solid tumors (used as a model for immune-evasive cancers such as NSCLC)	Demonstrated that NK cells are essential for the full therapeutic effect of PD-1/PD-L1 blockade. PD-1/PD-L1 blockade restored NK cytotoxicity and cytokine release, thereby expanding the role of checkpoint inhibition beyond T cells.	(53)
Multhoff et al. (55)	Randomized Phase II clinical trial in humans (unresectable stage IIIa/b NSCLC)	Autologous NK cells activated *ex vivo* with TKD peptide + IL-2, administered after radiochemotherapy vs. radiochemotherapy alone	Membrane Hsp70-positive advanced non-small cell lung cancer (NSCLC) after chemoradiotherapy	NK-cell therapy was well tolerated; 1-year progression-free survival estimate 67% in NK arm vs. 33% in control arm (though *P* = 0.36, 1-sided) and clinical responses correlated with increased activated NK cells in blood	(54)
Liu et al. (22)	Pre-clinical: *in vitro + in vivo* murine xenograft models of SCLC	CAR-engineered NK-92 cells targeting DLL3 (DLL3-CAR NK-92)	Small cell lung cancer (DLL3^+^ cell lines & mouse models)	Significantly enhanced NK cytotoxicity and cytokine production *in vitro*; tumor regression and inhibited growth *in vivo* with detectable CAR NK infiltration and no major toxicity.	(55)
Guevara Lopez et al. (57)	Pre-clinical; *in vitro* co-culture with NSCLC lines and 3D spheroids; *in vivo* patient-derived xenograft (PDX) mouse models	Cytokine-induced memory-like (CIML) NK cells, enriched in CD56^bright^ subset; tested alone and in combination with tri-specific NK engager (TriKE 1615133)	Non-small cell lung cancer (NSCLC), including cancer-stem-cell (CSC, CD133^+^) subpopulations	CIML-NK cells showed superior cytotoxicity and IFN-γ/CD107a secretion compared with conventional IL-2-activated NKs. The CD56^bright^ subset mediated strong anti-CSC activity and reduced tumorigenicity and lung dissemination in PDX models. Combination with TriKE 1615133 further enhanced killing of CD133^+^ CSCs, demonstrating a synergistic effect and highlighting CIML-NK/TriKE therapy as a promising next-generation approach in NSCLC.	(56)

Hsu et al. explored the contribution of NK cells to the antitumor effects of Programmed Cell Death Protein 1/Programmed Death-Ligand 1 (PD-1/PD-L1) blockade using several murine tumor models (53). Traditionally, checkpoint inhibitors have been viewed in the context of CD8^+^ T-cells, as these cytotoxic T cells recognize tumor antigens presented via MHC class I. However, clinical responses to PD-1/PD-L1 inhibitors have also been observed in tumors with low or absent MHC I expression, suggesting additional immune effector mechanisms (53). NK cells, which are known to eliminate target cells lacking MHC I, were therefore hypothesized to play a role. The study revealed that NK cells are indeed required for the full therapeutic effect of PD-1/PD-L1 blockade (53). In MHC I-deficient RMA-S tumor models, depletion of NK cells resulted in rapid tumor progression, whereas depletion of CD8^+^ T cells had little effect. Moreover, tumor-infiltrating NK cells were found to express PD-1, and their cytotoxic function was suppressed by PD-L1^+^ tumor cells *in vitro*. Importantly, anti-PD-1/PD-L1 therapy restored NK-cell degranulation and cytokine production, leading to improved tumor control. These findings expanded the understanding of checkpoint immunotherapy by identifying NK cells as direct targets of PD-1/PD-L1 inhibition, particularly relevant in tumors such as lung cancer, where MHC I downregulation commonly limits T-cell-mediated immunity (53).

Multhoff et al. focused on advanced non–small cell lung cancer (NSCLC) that tends to relapse even after adequate chemoradiotherapy (CRT) (54). One of the molecules, Heat Shock Protein 70 (Hsp70), is expressed on tumor cells that have undergone stress, such as after chemotherapy or radiotherapy. Based on Multhoff’s previous research, where they demonstrated that NK cells activated with a small fragment of Hsp70 (TKD peptide) plus IL-2 become highly cytotoxic against Hsp70^+^ tumor cells *in vitro*, this study aimed to translate those findings into a clinical setting. A total of 74 patients with unresectable stage IIIa/IIIb NSCLC who had completed standard CRT and showed no evidence of progression were recruited. The experimental arm received adoptive transfer of their own NK cells, which were collected from blood, expanded and activated in the lab with TKD + IL-2, and then reinfused. Results showed that NK-cell infusions were well tolerated. After 1 year, the progression-free survival (PFS) rate was 67% in the NK-therapy group compared to 33% in the control group. Furthermore, median overall survival (OS) trended higher in the NK-therapy group, though not statistically significant (*p value:0.36*; small sample size). Patients who responded clinically also exhibited increased levels of activated NK cells (CD56^bright^, CD94^+^/NKG2D^+^) in their peripheral blood (54).

**FIGURE 4:**
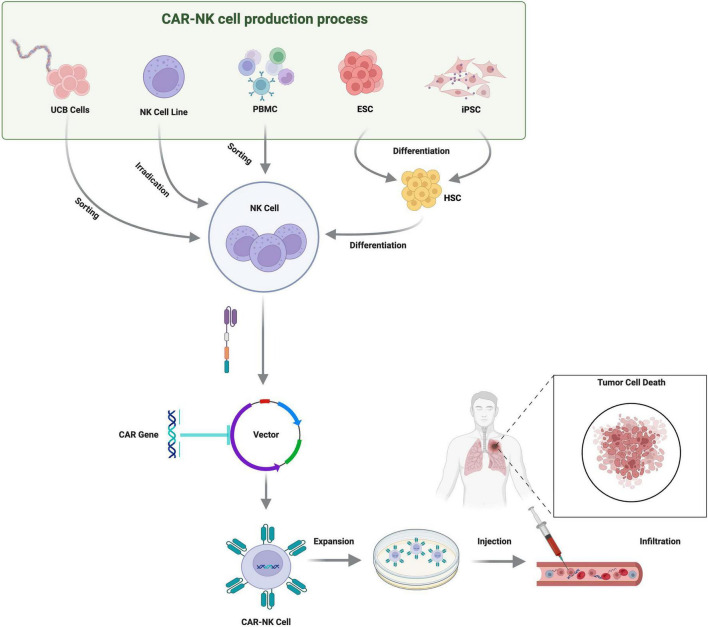
Overview of CAR-NK cell generation from diverse cell sources and their application in tumor targeting. This schematic outlines the production of CAR-engineered natural killer (CAR-NK) cells from multiple cellular sources, including umbilical cord blood, NK cell lines, peripheral blood mononuclear cells, embryonic stem cells, induced pluripotent stem cells, and hematopoietic stem cells. Following isolation or differentiation into NK cells, CAR constructs are introduced via viral or non-viral vectors, and the modified CAR-NK cells are subsequently expanded *ex vivo*. The final therapeutic product is administered to the patient, where CAR-NK cells infiltrate the tumor microenvironment and induce targeted tumor cell death.

Similarly, Liu et al. developed a chimeric antigen receptor (CAR) engineered NK cell line by modifying the NK-92 cell line to express a CAR targeting Delta-like ligand 3 (DLL3). DLL3 is a cell-surface protein that is highly overexpressed in small cell lung cancer (SCLC) but largely absent in normal adult tissues, making it an attractive and tumor-specific target. The study aimed to evaluate the antitumor efficacy of these DLL3-CAR NK-92 cells in both *in vitro* and *in vivo* models. *In vitro*, DLL3-CAR NK-92 cells exhibited significantly enhanced cytotoxicity and IFN-γ secretion when co-cultured with DLL3^+^ SCLC cell lines compared with unmodified NK-92 cells or DLL3^–^ tumor controls (55). Likewise, Guevara Lopez et al. investigated cytokine-induced memory-like (CIML) NK cells as a therapeutic strategy in non-small cell lung cancer (NSCLC). The study focused particularly on the CD56^bright^ NK-cell subset, which is known for its strong cytokine-producing capacity. CIML-NK cells significantly outperformed conventional IL-2-activated NK cells in killing NSCLC cell lines and 3D spheroids, showing higher IFN-γ secretion and CD107a degranulation. The study also demonstrated that the CD56^bright^ subset expanded markedly during CIML differentiation and acted as the primary effector population driving anti-cancer stem cell (CSC) activity, especially against CD133^+^ CSC subpopulations, in both spheroid cultures and patient-derived xenograft (PDX) models (56). An overview of CAR-NK cell generation from diverse sources and their application in tumor targeting is shown in Figure 4. Furthermore, while CAR-T and CAR-NK therapies share fundamental immunotherapeutic principles, they differ considerably in safety, *in vivo* persistence, manufacturing complexity, and clinical development stage. A concise comparative summary is provided in Table 3.

**TABLE 3:** Comparative overview of CAR-T and CAR-NK therapies in lung cancer.

Feature	CAR-T cells	CAR-NK cells
Safety	Risk of cytokine release syndrome (CRS) and neurotoxicity; off-tumor effects possible in lungs (26–28, 32, 41–44)	Lower CRS/neurotoxicity risk; selective killing via MHC-I recognition reduces off-tumor toxicity (45, 46, 54–56)
Persistence	Limited in solid tumors; enhanced by armored CARs (IL-12, IL-18), chemokine receptor co-expression, and logic-gated designs, but clinical persistence remains short (29–31, 36, 41–44)	Naturally shorter-lived; improved persistence demonstrated by cytokine-induced memory-like NK cells (CIML-NK) and IL-2/TKD activation (54–56)
Manufacturing complexity	Autologous production; labor-intensive, patient-specific; allogeneic “off-the-shelf” CAR-T under investigation to reduce turnaround (35, 40)	Off-the-shelf potential; scalable from NK-92 cell lines, peripheral blood, or cord blood; simpler manufacturing (45, 55, 56)
Clinical maturity	Advanced; multiple early-phase trials in NSCLC and SCLC; strong mechanistic and translational data (28, 41–44)	Early clinical evaluation; promising preclinical efficacy; limited but growing human data (54–56)

## Advances in TIL therapy in lung cancer

Researchers observed that a high density of tumor-infiltrating lymphocytes (TILs) correlated with favorable clinical outcomes in patients with melanoma and colorectal cancer (CRC). Since then, a substantial body of evidence has demonstrated the prognostic significance of TIL abundance across multiple solid tumors. Pathologist-based scoring systems for TIL density have even been proposed as tools for risk stratification in certain cancers, such as melanoma, breast cancer, and colorectal cancer (57).

TILs primarily comprise a heterogeneous population of αβ T cells, both CD4^+^ helper and CD8^+^ cytotoxic subsets, within the TME. However, the immune infiltrate of human tumors is far more complex, consisting of diverse hematopoietic and stromal components, including macrophages (M0/M1/M2), dendritic cells, B cells, NK cells, mast cells, γδ T cells, and variable populations of neutrophils and eosinophils. Despite this complexity, studies have consistently shown that the composition, localization, and functional state of TILs critically influence tumor progression and therapeutic response (58).

The recent U.S. Food and Drug Administration (FDA) approval of Lifileucel (Amtagvi, Iovance Biotherapeutics) for patients with unresectable or metastatic melanoma who have previously received immune checkpoint inhibitors (ICIs) or BRAF inhibitors has significantly bolstered enthusiasm for extending TIL therapy to other solid malignancies, including lung cancer (59). TIL therapy involves isolating lymphocytes from surgically resected or biopsied tumors, expanding them *ex vivo*, and reinfusing them into the patient after lymphodepleting conditioning. The resulting infusion product, comprising a diverse repertoire of polyclonal, tumor-reactive T cells, offers key advantages, such as broad tumor antigen recognition, minimal off-target toxicity, and intrinsic tumor-homing capacity (60).

**FIGURE 5:**
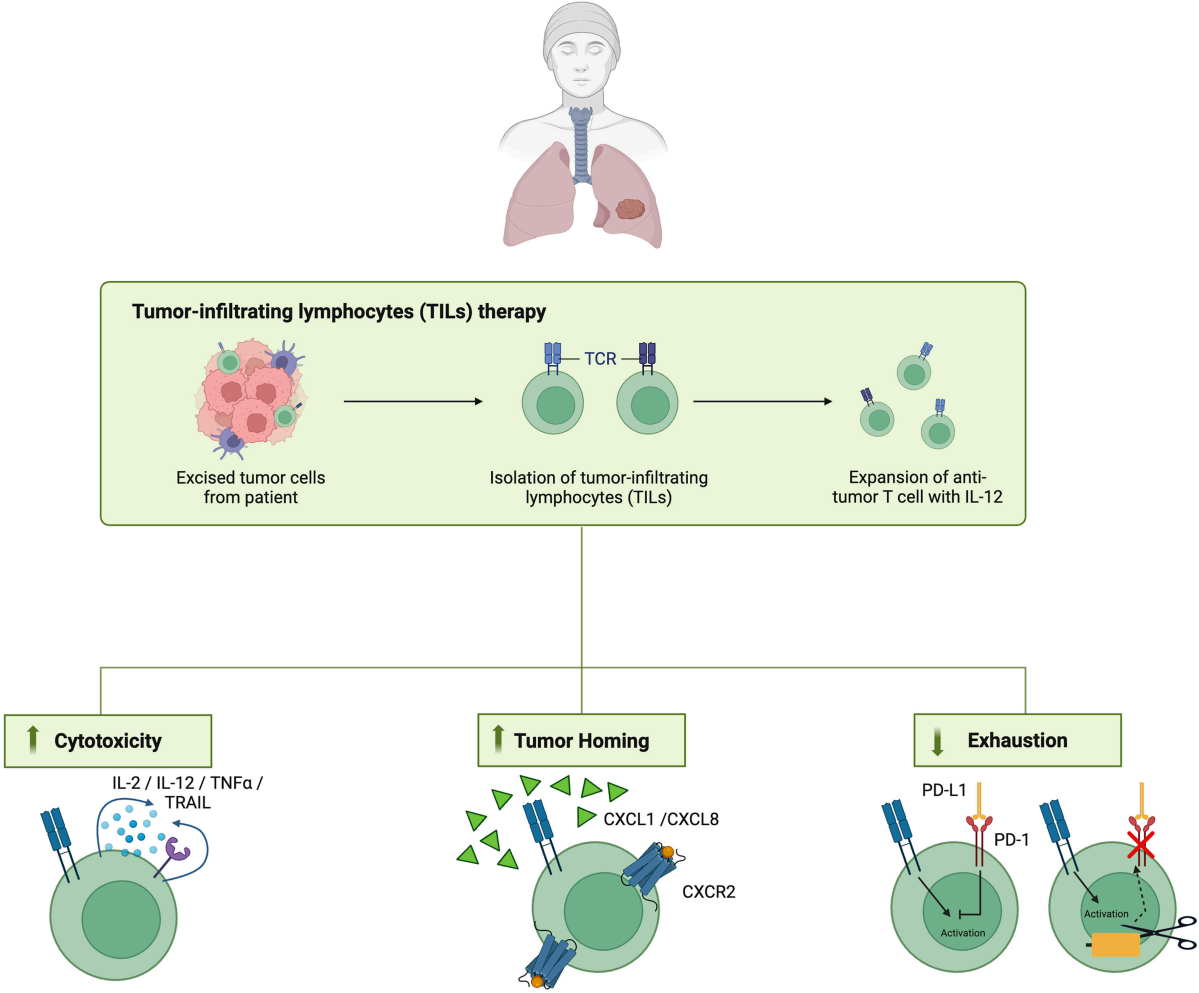
Engineering strategies to enhance tumor-infiltrating lymphocyte (TIL) therapy. The figure outlines the workflow of TIL therapy, from the isolation of TILs from resected tumor tissue to *ex vivo* expansion, and highlights engineering strategies to improve therapeutic efficacy. Approaches include boosting cytotoxicity through cytokine support, enhancing tumor homing via chemokine receptor expression of chemokine receptors, and reducing T-cell exhaustion by disrupting inhibitory pathways, such as the PD-1/PD-L1 pathway.

Unlike engineered CAR-T or TCR-T cells, TILs naturally comprise of diverse T cell clones that can recognize multiple tumor antigens, including neoantigens, and thus can bypass central tolerance. Importantly, TIL therapy has not been associated with off-tumor, on-target (OTOT) or cytokine release syndrome (CRS) toxicities, complications often seen with CAR-T therapy. Additionally, because TILs are obtained from the patient’s tumor tissue, they are inherently equipped with tumor-homing chemokine receptors (e.g., CX3CR1 and CCR4). The presence of such receptors guides the cells to the tumor. Given these characteristics, TIL therapy may represent a potential next-generation immunotherapeutic approach for solid tumors, including lung cancer (61) (Figure 5).

Ben-Avi et al. discuss the practicality of generating tumor-infiltrating lymphocytes (TILs) from non-small cell lung cancer tissue. Tumor samples were taken from 5 patients with diagnosed NSCLC. There were successful TIL cultures produced, and they displayed activation markers such as PD-1, CD28, and 4-1BB. These TILs contained a higher proportion of CD4^+^ T cells, and some showed tumor-specific cytotoxicity against autologous lung cancer cell lines, indicating genuine antitumor reactivity. Even though this study was preclinical and did not involve infusion back into the patient or outcome data, it did prove that NSCLC tumors can be a viable source of expandable TILs (62). The recent advancements in TIL-based adoptive cell therapy for lung cancer are summarized in Table 4. Furthermore, while TIL therapy offers advantages such as inherent tumor homing and a reduced risk of cytokine release syndrome (CRS), several practical and biological limitations must be considered. The efficacy of TIL therapy is highly dependent on the availability of viable tumor tissue, which may be limited in small, necrotic, or inaccessible lung tumors. (61) Manufacturing timelines are also a significant constraint, as *ex vivo* expansion of patient-derived TILs can take several weeks, during which disease progression may occur (61). Additionally, preparative regimens involving lymphodepletion and high-dose IL-2 administration can introduce toxicity, limiting eligibility for patients with compromised performance status (58, 61).

**TABLE 4:** Clinical and pre-clinical investigations of tumor-infiltrating lymphocyte (TIL)–based therapies in non-small cell lung cancer.

Study type/phase	Study type/phase	Tumor context/population	TIL approach/treatment strategy	Main or key findings	References
Ben-Avi et al. (62) *Cancer Immunology, Immunotherapy*	Preclinical feasibility study	Non-Small Cell Lung Cancer (NSCLC)	Isolation and ex vivo expansion of TILs from surgical NSCLC samples (*n* = 5); compared with 103 melanoma-derived TIL cultures	TILs successfully expanded from all NSCLC tumors; growth comparable to melanoma TILs; higher CD4^+^ fraction; expressed PD-1, CD28, 4-1BB; two TIL cultures showed autologous tumor-specific cytotoxicity.	(62)
NCT03215810 (49)	Phase I, single-arm, open-label	Metastatic/advanced NSCLC patients refractory to PD-1 blockade	Patients first received nivolumab; metastatic lesion surgically resected to harvest TILs; TILs expanded ex vivo; lymphodepletion; TIL infusion + high-dose IL-2; maintenance nivolumab	Feasibility and safety demonstrated; objective tumor regressions observed (including complete responses lasting > 1.5 years); ∼20% overall response rate; first proof-of-concept that TIL ACT can work in checkpoint-resistant metastatic NSCLC.	(70)
NCI-2017-01966 (50)	Pilot Phase I	Recurrent or stage IV NSCLC (including post-therapy recurrence)	Combination: lymphodepleting chemo (cyclophosphamide + fludarabine), nivolumab (anti–PD-1), infusion of autologous expanded TILs, plus high-dose IL-2 support	Showed safety and biological activity; multimodal combination (chemo, PD-1 blockade, and cytokine support) is clinically feasible in advanced NSCLC.	(71)
IOV-LUN-202 (51)	Phase II, multicenter	Metastatic/unresectable NSCLC after chemo-immunotherapy failure	One-time infusion of autologous TIL product (LN-145/lifileucel-like) after lymphodepletion; no engineered CAR/TCR (pure TIL ACT)	Demonstrated feasibility, safety, and > 20% objective response rate; indicates durable responses possible post–PD-1/PD-L1 failure.	(72)
NCT05902520 (52)	Phase I, first-in-human	Advanced solid tumors (including NSCLC cohorts)	Enrichment of a specific CD8^+^ TIL subpopulation, highly tumor-reactive (AGX148), expanded and reinfused	Evaluates “smart-selected” precision-TILs instead of bulk TILs; NSCLC explicitly included in basket trial.	(73)
NCT06455917 (53)	Phase I (ongoing)	NSCLC patients progressing on immune checkpoint inhibitors	Autologous TIL therapy following ex vivo expansion and lymphodepletion	Early-phase evaluation of TIL ACT efficacy in ICI-resistant NSCLC; focuses on safety and initial antitumor activity.	(74)

TIL, tumor-infiltrating lymphocyte; NSCLC, non-small cell lung cancer; IL-2, interleukin-2; ACT, adoptive cell therapy; ICI, immune checkpoint inhibitor.

## Key Challenges and future directions

Despite significant advancements, next-generation immune cell therapies for lung cancer, including CAR-T, CAR-NK, and TIL therapies, face substantial biological, technical, and translational challenges that must be addressed to achieve durable and safe clinical efficacy. The most critical challenges and corresponding future directions are summarized below:

## Antigen selection, heterogeneity, and antigen escape

A major challenge in adoptive cell therapy for lung cancer is the inherent diversity of tumor cells and the tendency of some to lose the antigens targeted by treatment. Most current CAR-T and CAR-NK approaches focus on single antigens such as mesothelin, EGFR, MUC1, ROR1, or DLL3, which are not uniformly expressed across all tumor cells and may also be present at low levels in normal lung tissue, increasing the risk of on-target/off-tumor (OTOT) toxicity (63, 64). A recent review by Kandra et al. (65), further outlines these challenges in CAR-T therapy for lung cancer, including antigen heterogeneity, limited persistence, and the need for combinatorial or multi-antigen strategies to overcome tumor escape and improve safety. (65) Several strategies are being explored to enhance the selectivity of CAR-based therapies. Multi-antigen targeting or logic-gated CAR designs, which require the engagement of two antigens (AND gate) or incorporate inhibitory modules when a normal-cell antigen is detected (NOT gate), ensure that CARs selectively target tumor cells while sparing healthy tissue (63, 66). Affinity-tuned Single-chain variable fragments (scFvs) that bind more weakly to antigens expressed on normal cells but strongly to tumor-associated antigens and glycoform-specific binders (e.g., MUC1 glycovariants) can further improve precision (64). Modular or universal CAR systems with switchable adapters are emerging as versatile solutions that allow real-time retargeting and safety control without the need for new manufacturing runs (67).

## Tumor microenvironment immunosuppression and infiltration barriers

The lung tumor microenvironment (TME) poses multiple barriers to adoptive cell therapy by creating conditions that are hostile to immune cells. Hypoxia impairs cellular metabolism, and fibrosis forms dense tissue that restricts infiltration, and immunosuppressive cytokines, such as TGF-β and IL-10, along with adenosine accumulation, suppress cytotoxic activity. Inhibitory checkpoint ligands, such as PD-L1, further limit immune recognition, collectively reducing the trafficking and persistence of therapeutic cells (63, 66, 68). To overcome the suppressive tumor microenvironment, armored cell therapies are engineered to secrete proinflammatory cytokines such as IL-12 or IL-18, which locally counteract immunosuppressive signals, enhance the activation and persistence of immune cells, and restore their cytotoxic function against tumor cells. (66, 68). Co-expressing chemokine receptors, such as CXCR1/2 and CCR2/5, on adoptively transferred immune cells enhances their ability to follow chemical signals released by tumors, thereby improving migration and accumulation at the tumor site. These cells can be delivered either into the tumor or near it through intratumoral or intrapleural injections. This not only helps in concentrating the immune cells in regions where they are needed but also prevents systemic side effects, making the treatment effective and safe (64, 68). It is expected that adoptive cell therapy CAR-T, CAR-NK, or TILs) will be combined with additional treatments to increase its effectiveness. Examples of this supportive approach are Immune checkpoint inhibitor drugs, Oncolytic viruses, which may be genetically engineered and used to stimulate the immune system against the tumor, Metabolic therapies that target the metabolism of cancer cells, making them weaker and more vulnerable to attack by adoptive cell therapy (63, 66).

## Persistence and durability of effect

Donor-derived CAR-T cells and CAR-engineered NK cells often fail to persist long term *in vivo*, a limitation that contributes to suboptimal tumor control, emerging strategies such as CISH deletion in human iPSC-derived NK cells have demonstrated markedly improved persistence and enhanced antitumor efficacy through metabolic reprogramming, which may help overcome this barrier. (69) This leads to weaker or temporary tumor control compared to autologous CAR-T cells, which come from the patient and naturally persist longer. Scientists genetically modify these cells, making them able to produce molecules that help them grow and last longer inside the patient. Examples of such molecules are IL-15 or IL-15 with its receptor IL-15Ra. Other strategies include using a special type of NK cell called Cytokine-induced memory -like (CIML), which is exposed to a certain cytokine before it is given to the patient, and this makes it behave more like a memory cell, resulting in a long-lasting response. Tri-specific killer engagers (TriKEs) are designed to help NK cells find and attack the tumor cells more precisely (55, 66).

## Safety and toxicity

CAR-T Therapy triggers strong immune reactions like Cytokine release syndrome, causing dangerous symptoms like low blood pressure and organ failure, neurotoxicity, which may lead to confusion and seizures, and on-target off-tumor effects, which damage the normal cells. CAR-NK and TIL therapies have a more controlled immune response, making them safer; however, the pre-treatment regimens like Lymphodepletion or IL-2 administration are toxic on their own, and not all patients are healthy enough to tolerate which limits the eligibility of patients who can receive treatment (57, 66). To make these therapies safer, the engineered cells include a self-destructive switch, such as inducible caspase-9 (iCasp9), which can be activated in case of a severe adverse reaction, causing the therapeutic cells to die and stop the toxic reaction. To prevent healthy cells from being attacked, two engineering strategies are used, which include Affinity-tuned CARs that only activate when antigen levels are very high, such as in tumors, and not when low, such as in normal cells. Dual-antigen CARs require two specific antigens to be present before getting activated (27, 64, 66).

## Manufacturing, scalability, and accessibility

Production and delivery of Adoptive cell treatments is difficult. Autologous therapies are time-consuming, labor-intensive, and also expensive. Since lung cancer progresses rapidly, the patient does not have enough time to wait for this process. For TIL therapy, in order to grow TIL, a large amount of healthy and living tumor tissue is required, which sometimes becomes difficult for the doctor to collect, either because the tumor is too small, inaccessible, or the biopsy does not yield enough viable cells. Even though Allogeneic cells and iPSC-derived cells can be manufactured in advance and used immediately like an off-the-shelf medicine, there is a risk of immune rejection. To improve ACT, we need simpler, faster, and larger-scale methods for making these cells. (63, 66, 67).

## Limited clinical data and biomarker uncertainty

Most evidence for these therapies in lung cancer comes from lab studies or early-phase trials. The studies show mixed or inconsistent response rates; hence, the efficacy has not been clearly established (57, 63, 67). To choose the right patient for these therapies, reliable biomarkers are needed. These biomarkers include antigen density on the tumor cells, tumor mutational burden, Infiltration profile of T-cells, and Immune checkpoint expression (13). Future research should use more advanced methods like adaptive trial designs, real-time molecular imaging to track these cells inside the body, Integrated Immune monitoring, etc. This will help the researchers understand the persistence, trafficking, and response of this therapy (63).

## Tumor escape, resistance, and combination strategies

Lung tumors have several ways to escape ACT, including loss of target antigens as they stop expressing the proteins that CAR-T or CAR-NK cells are designed to recognize. Shedding of ligands (e.g., soluble NKG2D ligands), which are molecules released into the bloodstream that bind to NK or CAR-NK receptors and weaken their ability to kill. Metabolic suppression, where tumors create a hostile metabolic environment by producing adenosine, which suppresses T cells and lactate that lowers pH and hence inhibits immune cells. This weakens ACT cells in the tumor microenvironment. There is upregulation of inhibitory checkpoint molecules like PD-L1 that shut down T cells and NK cells. To overcome these barriers, ACT can be combined with metabolic modulators such as Adenosine A2A receptor antagonists that block adenosine-mediated inhibition and Indoleamine 2,3-dioxygenase (IDO) inhibitors that prevent tryptophan depletion. Genetically modified immune cells ignore tumor-induced inhibitory signals. For example, they develop resistance to TGF-β, a powerful immunosuppressive molecule in lung tumors. Additional strategies aim to make tumors more visible and responsive to immune attacks. This is done by converting them from cold to hot tumors, as hot tumors have high immune infiltration. The methods used to convert include Radiotherapy, which releases tumor antigens, increasing inflammation, Stimulator of Interferon Genes (STING) agonists that activate the innate immune pathways, attracting T-cells, and Oncolytic viruses that infect and kill tumor cells, stimulating a strong immune response (63, 66, 68).

## CAR-NK–specific technical barriers

CAR-NK therapies are safer than CAR-T; however, they still face important challenges. NK cells are harder to genetically modify because they resist viral transduction, making CAR insertion less efficient. Their performance also varies depending on the cell source, which can be the peripheral blood, cord blood, iPSCs, and NK-92. All differ in expansion capacity, cytotoxic strength, and persistence. NK cells typically have a short lifespan in the body after infusion, which limits durable tumor control. To address these challenges, the field needs reliable and consistent ways to manufacture NK cells, especially those derived from iPSCs, which can be produced at a large scale and stored as off-the-shelf products, and from cord blood, which offers a dependable and expandable source. Creating standardized processes helps ensure that each batch behaves the way it’s supposed to, making the therapies more predictable, reproducible, and clinically dependable. CIML NK cells are generated by exposing NK cells to cytokines such as IL-12, IL-15, and IL-18. This increases their strength and not only helps them remain active for longer but also makes them more responsive when they encounter tumor cells (55, 66).

## Regulatory, ethical, and accessibility issues

Regulatory complexity surrounding gene-edited and iPSC-derived cellular products, along with high costs, limit widespread implementation (18, 68). Harmonizing potency testing, toxicity reporting, and cost-reduction strategies through early engagement with regulatory bodies will be essential. Moreover, global collaboration and standardized safety criteria are needed to ensure equitable patient access and the safe integration of these therapies into lung-cancer management (57, 66). Gene-edited and iPSC-derived therapies are heavily regulated because they involve genetic modifications and stem-cell technologies. These regulations, combined with high production costs, make it difficult to implement these therapies on a large scale (18, 68). To make these therapies more accessible, developers need to align testing standards and reporting methods with regulators early in the process.

## Conclusion

Advances in CAR-T, CAR-NK, and TIL therapies are rapidly expanding the therapeutic landscape for lung cancer; however, clinical benefit remains limited due to antigen heterogeneity, immune evasion, a suppressive tumor microenvironment, and insufficient persistence of transferred cells. Emerging strategies such as multi-antigen targeting, armored and logic-gated constructs, regional delivery, and combination approaches with checkpoint blockade or targeted agents show promise in improving safety and antitumor activity. CAR-NK therapies have the advantage of being safer and easier to produce as off-the-shelf treatments, while TIL therapies have shown feasibility even in patients who no longer respond to PD-1 inhibitors, especially when enriched for tumor-specific neoantigens. Improving the persistence of these cells, scaling up manufacturing, and making them resistant to exhaustion will be critical to turning these therapies into durable treatments. Overall, next-generation immune cell therapies are getting closer to routine clinical use in lung cancer, and ongoing innovations are expected to make responses more consistent and long-lasting.

## References

[1] Wéber A Morgan E Vignat J Laversanne M Pizzato M Rumgay H Lung cancer mortality in the wake of the changing smoking epidemic: a descriptive study of the global burden in 2020 and 2040. *BMJ Open.* (2023) 13:e065303. 10.1136/bmjopen-2022-065303 37164477 PMC10174019

[2] Travis WD Brambilla E Nicholson AG Yatabe Y Austin JHM Beasley MB The 2015 World Health Organization classification of lung tumors: impact of genetic, clinical and radiologic advances since the 2004 classification. *J Thorac Oncol.* (2015) 10:1243–60. 10.1097/JTO.0000000000000630 26291008

[3] Bi JH Tuo JY Xiao YX Tang DD Zhou XH Jiang YF Observed and relative survival trends of lung cancer: a systematic review of population-based cancer registration data. *Thorac Cancer.* (2024) 15:142–51. 10.1111/1759-7714.15170 37986711 PMC10788469

[4] Us Preventive Services Task Force, Krist AH Davidson KW Mangione CM Barry MJ Cabana M Screening for lung Cancer: US preventive services task force recommendation statement. *JAMA.* (2021) 325:962–70. 10.1001/jama.2021.1117 33687470

[5] Pinsky PF. Assessing the benefits and harms of low-dose computed tomography screening for lung cancer. *Lung Cancer Manag.* (2014) 3:491–8. 10.2217/LMT.14.41 26617677 PMC4662564

[6] La’ah AS Chiou SH. Cutting-Edge therapies for lung Cancer. *Cells.* (2024) 13:436. 10.3390/cells13050436 38474400 PMC10930724

[7] Zappa C Mousa SA. Non-small cell lung cancer: current treatment and future advances. *Transl Lung Cancer Res.* (2016) 5:288–300. 10.21037/tlcr.2016.06.07 27413711 PMC4931124

[8] Gandhi L Rodríguez-Abreu D Gadgeel S Esteban E Felip E De Angelis F Pembrolizumab plus chemotherapy in metastatic non-small-cell Lung Cancer. *N Engl J Med.* (2018) 378:2078–92. 10.1056/NEJMoa1801005 29658856

[9] Sharma P Allison JP. The future of immune checkpoint therapy. *Science.* (2015) 348:56–61. 10.1126/science.aaa8172 25838373

[10] DiNofia AM Maude SL. Chimeric antigen receptor T-Cell therapy clinical results in pediatric and young adult B-ALL. *Hemasphere.* (2019) 3:e279. 10.1097/HS9.0000000000000279 31723849 PMC6745916

[11] Cha D Lim C-M Kwack K Park JE. Emerging advances in solid tumor immunotherapy: a comparative review of CAR-T, CAR-NK, CAR-M, and TIL therapies. *Yakhak Hoeji.* (2025) 69:212–23. 10.17480/psk.2025.69.3.212

[12] Vo MC Tran VD Nguyen VT Ruzimurodov N Trung DT Kim SK Challenges and limitations of chimeric antigen receptor T-cell therapies in solid tumors: why are approvals restricted to hematologic malignancies? *J Hematol Oncol.* (2025) 18:91. 10.1186/s13045-025-01744-9 41146230 PMC12557894

[13] Ma HY Das J Prendergast C De Jong D Braumuller B Paily J Advances in CAR T cell therapy for non-small cell Lung Cancer. *Curr Issues Mol Biol.* (2023) 45:9019–38. 10.3390/cimb45110566 37998743 PMC10670348

[14] Zhang Z Jiang J Wu X Zhang M Luo D Zhang R Chimeric antigen receptor T cell targeting EGFRvIII for metastatic lung cancer therapy. *Front Med.* (2019) 13:57–68. 10.1007/s11684-019-0683-y 30721445

[15] Wei X Lai Y Li J Qin L Xu Y Zhao R PSCA and MUC1 in non-small-cell lung cancer as targets of chimeric antigen receptor T cells. *Oncoimmunology.* (2017) 6:e1284722. 10.1080/2162402X.2017.1284722 28405515 PMC5384358

[16] Brannan JM Dong W Prudkin L Behrens C Lotan R Bekele BN Expression of the receptor tyrosine kinase EphA2 is increased in smokers and predicts poor survival in non-small cell lung cancer. *Clin Cancer Res.* (2009) 15:4423–30. 10.1158/1078-0432.CCR-09-0473 19531623

[17] Sanomachi T Katsuya Y Nakatsura T Koyama T. Next-Generation CAR-T and TCR-T cell therapies for solid tumors: innovations, challenges, and global development trends. *Cancers.* (2025) 17:1945. 10.3390/cancers17121945 40563595 PMC12191048

[18] Page A Chuvin N Valladeau-Guilemond J Depil S. Development of NK cell-based cancer immunotherapies through receptor engineering. *Cell Mol Immunol.* (2024) 21:315–31. 10.1038/s41423-024-01145-x 38443448 PMC10978891

[19] Zhang F Soleimani Samarkhazan H Pooraskari Z Bayani A. Beyond CAR-T: engineered NK cell therapies (CAR-NK, NKCEs) in next-generation cancer immunotherapy. *Crit Rev Oncol Hematol.* (2025) 214:104912. 10.1016/j.critrevonc.2025.104912 40848820

[20] Kumar A Fischer C Cichocki F Miller JS. Multiplexed iPSC platform for advanced NK cell immunotherapies. *Cell Rep Med.* (2025) 6:102282. 10.1016/j.xcrm.2025.102282 40780202 PMC12711691

[21] Hu W Bian Y Ji H. TIL therapy in Lung Cancer: current progress and perspectives. *Adv Sci.* (2024) 11:e2409356. 10.1002/advs.202409356 39422665 PMC11633538

[22] Amaria RN Komanduri KV Schoenfeld AJ Ramsingh G Burga RA Jagasia MH. Entering a new era of tumor-infiltrating lymphocyte cell therapy innovation. *Cytotherapy.* (2025) 27:864–73. 10.1016/j.jcyt.2024.12.010 40131263

[23] Abodunrin F Olson DJ Emehinola O Bestvina CM. Adopting tomorrow’s therapies today: a perspective review of adoptive cell therapy in lung cancer. *Ther Adv Med Oncol.* (2025) 17:17588359251320280. 10.1177/17588359251320280 40012708 PMC11863254

[24] Zhong S Cui Y Liu Q Chen S. CAR-T cell therapy for lung cancer: a promising but challenging future. *J Thorac Dis.* (2020) 12:4516–21. 10.21037/jtd.2020.03.118 32944366 PMC7475572

[25] Xu C Ju D Zhang X. Chimeric antigen receptor T-cell therapy: challenges and opportunities in lung cancer. *Antibody Therapeut.* (2022) 5:73–83. 10.1093/abt/tbac006 35372786 PMC8972219

[26] Flugel CL Majzner RG Krenciute G Dotti G Riddell SR Wagner DL Overcoming on-target, off-tumour toxicity of CAR T cell therapy for solid tumours. *Nat Rev Clin Oncol.* (2023) 20:49–62. 10.1038/s41571-022-00704-3 36418477 PMC10278599

[27] Yang Y Vedvyas Y Alcaina Y Trumper SJ Babu DS Min IM Affinity-tuned mesothelin CAR T cells demonstrate enhanced targeting specificity and reduced off-tumor toxicity. *JCI Insight.* (2024) 9:e186268. 10.1172/jci.insight.186268 39576012 PMC11601908

[28] Adusumilli PS Zauderer MG Rivière I Solomon SB Rusch VW O’Cearbhaill RE A Phase I trial of regional mesothelin-targeted CAR T-cell therapy in patients with malignant pleural disease, in combination with the Anti-PD-1 agent pembrolizumab. *Cancer Discov.* (2021) 11:2748–63. 10.1158/2159-8290.CD-21-0407 34266984 PMC8563385

[29] Andreou T Neophytou C Kalli M Mpekris F Stylianopoulos T. Breaking barriers: enhancing CAR-armored T cell therapy for solid tumors through microenvironment remodeling. *Front Immunol.* (2025) 16:1638186. 10.3389/fimmu.2025.1638186 40969762 PMC12441034

[30] Jaspers JE Khan JF Godfrey WD Lopez AV Ciampricotti M Rudin CM IL-18-secreting CAR T cells targeting DLL3 are highly effective in small cell lung cancer models. *J Clin Invest.* (2023) 133:e166028. 10.1172/JCI166028 36951942 PMC10145930

[31] Hu X Ge C Huang C He D Yao X Cheng J Enhanced homing and efficacy of HER2-CAR T cells via CXCR5/CCR6 co-expression for HER2-positive NSCLC. *J Transl Med.* (2025) 23:863. 10.1186/s12967-025-06866-9 40764989 PMC12326854

[32] Azeez SS Yashooa RK Smail SW Salihi A Ali AS Mamand S Advancing CAR-based cell therapies for solid tumours: challenges, therapeutic strategies, and perspectives. *Mol Cancer.* (2025) 24:191. 10.1186/s12943-025-02386-8 40624498 PMC12232864

[33] Adusumilli PS Cherkassky L Villena-Vargas J Colovos C Servais E Plotkin J Regional delivery of mesothelin-targeted CAR T cell therapy generates potent and long-lasting CD4-dependent tumor immunity. *Sci Transl Med.* (2014) 6:261ra151. 10.1126/scitranslmed.3010162 25378643 PMC4373413

[34] Zheng W Zhu T Tang L Li Z Jiang G Huang X. Inhalable CAR-T cell-derived exosomes as paclitaxel carriers for treating lung cancer. *J Transl Med.* (2023) 21:383. 10.1186/s12967-023-04206-3 37308954 PMC10262566

[35] Shokati A Sanjari-Pour M Akhavan Rahnama M Hoseinzadeh S Vaezi M Ahmadvand M. Allogeneic CART progress: platforms, current progress and limitations. *Front Immunol.* (2025) 16:1557157. 10.3389/fimmu.2025.1557157 40574859 PMC12198129

[36] Clinicaltrials.gov. Available online at: https://clinicaltrials.gov/study/NCT06051695. (2025). (accessed November 15, 2025).

[37] Maalej KM Merhi M Inchakalody VP Mestiri S Alam M Maccalli C CAR-cell therapy in the era of solid tumor treatment: current challenges and emerging therapeutic advances. *Mol Cancer.* (2023) 22:20. 10.1186/s12943-023-01723-z 36717905 PMC9885707

[38] Hovhannisyan L Riether C Aebersold DM Medová M Zimmer Y. CAR T cell-based immunotherapy and radiation therapy: potential, promises and risks. *Mol Cancer.* (2023) 22:82. 10.1186/s12943-023-01775-1 37173782 PMC10176707

[39] Rezaei R Esmaeili Gouvarchin Ghaleh H Farzanehpour M Dorostkar R Ranjbar R Bolandian M Combination therapy with CAR T cells and oncolytic viruses: a new era in cancer immunotherapy. *Cancer Gene Ther.* (2022) 29:647–60. 10.1038/s41417-021-00359-9 34158626

[40] Rafii S Mukherji D Komaranchath AS Khalil C Iqbal F Abdelwahab SI Advancing CAR T-Cell therapy in solid tumors: current landscape and future directions. *Cancers.* (2025) 17:2898. 10.3390/cancers17172898 40940995 PMC12428560

[41] Lin Y Chen S Zhong S An H Yin H McGowan E. Phase I clinical trial of PD-1 knockout anti-MUC1 CAR-T cells in the treatment of patients with non-small cell lung cancer. *Ann Oncol.* (2019) 30:xi12. 10.1093/annonc/mdz448

[42] Zhou D Byers LA Sable B Smit MD Sadraei NH Dutta S Clinical pharmacology profile of AMG 119, the first Chimeric Antigen Receptor T (CAR-T) Cell Therapy Targeting Delta-Like Ligand 3 (DLL3), in Patients with Relapsed/Refractory Small Cell Lung Cancer (SCLC). *J Clin Pharmacol.* (2024) 64:362–70. 10.1002/jcph.2346 37694295

[43] Zhang Y Zhang Z Ding Y Fang Y Wang P Chu W Phase I clinical trial of EGFR-specific CAR-T cells generated by the piggyBac transposon system in advanced relapsed/refractory non-small cell lung cancer patients. *J Cancer Res Clin Oncol.* (2021) 147:3725–34. 10.1007/s00432-021-03613-7 34032893 PMC11801842

[44] Clinicaltrials.gov. Available online at: https://clinicaltrials.gov/study/NCT02706392. (2025). (accessed December 10, 2025).

[45] Abel AM Yang C Thakar MS Malarkannan S. Natural killer cells: development, maturation, and clinical utilization. *Front Immunol.* (2018) 9:1869. 10.3389/fimmu.2018.01869 30150991 PMC6099181

[46] Chan CJ Smyth MJ Martinet L. Molecular mechanisms of natural killer cell activation in response to cellular stress. *Cell Death Differ.* (2014) 21:5–14. 10.1038/cdd.2013.26 23579243 PMC3857624

[47] Imai K Matsuyama S Miyake S Suga K Nakachi K. Natural cytotoxic activity of peripheral-blood lymphocytes and cancer incidence: an 11-year follow-up study of a general population. *Lancet.* (2000) 356:1795–9. 10.1016/S0140-6736(00)03231-1 11117911

[48] Viel S Besson L Marotel M Walzer T Marçais A. Regulation of mTOR, metabolic fitness, and effector functions by cytokines in natural killer cells. *Cancers.* (2017) 9:132. 10.3390/cancers9100132 28956813 PMC5664071

[49] Young A Ngiow SF Gao Y Patch AM Barkauskas DS Messaoudene M A2AR adenosine signaling suppresses natural killer cell maturation in the tumor microenvironment. *Cancer Res.* (2018) 78:1003–16. 10.1158/0008-5472.CAN-17-2826 29229601

[50] Harmon C Robinson MW Hand F Almuaili D Mentor K Houlihan DD Lactate-Mediated acidification of tumor microenvironment induces apoptosis of liver-resident NK cells in colorectal liver metastasis. *Cancer Immunol Res.* (2019) 7:335–46. 10.1158/2326-6066.CIR-18-0481 30563827

[51] Fernández-Messina L Ashiru O Boutet P Agüera-González S Skepper JN Reyburn HT Differential mechanisms of shedding of the glycosylphosphatidylinositol (GPI)-anchored NKG2D ligands. *J Biol Chem.* (2010) 285:8543–51. 10.1074/jbc.M109.045906 20080967 PMC2838276

[52] Hess JB Sutherland KD Best SA. Exploring natural killer cell immunology as a therapeutic strategy in lung cancer. *Transl Lung Cancer Res.* (2021) 10:2788–805. 10.21037/tlcr-20-765 34295678 PMC8264324

[53] Zingoni A Fionda C Borrelli C Cippitelli M Santoni A Soriani A. Natural killer cell response to chemotherapy-stressed cancer cells: role in tumor immunosurveillance. *Front Immunol.* (2017) 8:1194. 10.3389/fimmu.2017.01194 28993779 PMC5622151

[54] Hsu J Hodgins JJ Marathe M Nicolai CJ Bourgeois-Daigneault MC Trevino TN Contribution of NK cells to immunotherapy mediated by PD-1/PD-L1 blockade. *J Clin Invest.* (2018) 128:4654–68. 10.1172/JCI99317 30198904 PMC6159991

[55] Multhoff G Seier S Stangl S Sievert W Shevtsov M Werner C Targeted natural killer cell-based adoptive immunotherapy for the treatment of patients with NSCLC after radiochemotherapy: a randomized Phase II clinical trial. *Clin Cancer Res.* (2020) 26:5368–79. 10.1158/1078-0432.CCR-20-1141 32873573

[56] Liu M Huang W Guo Y Zhou Y Zhi C Chen J CAR NK-92 cells targeting DLL3 kill effectively small cell lung cancer cells in vitro and in vivo. *J Leukoc Biol.* (2022) 112:901–11. 10.1002/JLB.5MA0122-467R 35088475

[57] Guevara Lopez ML Gebo A Parodi M Persano S Maus-Conn J Mingari MC CD56bright cytokine-induced memory-like NK cells and NK-cell engagers synergize against non-small cell lung cancer cancer-stem cells. *J Immunother Cancer.* (2025) 13:e010205. 10.1136/jitc-2024-010205 39939140 PMC11822435

[58] Lopez de Rodas M Villalba-Esparza M Sanmamed MF Chen L Rimm DL Schalper KA. Biological and clinical significance of tumour-infiltrating lymphocytes in the era of immunotherapy: a multidimensional approach. *Nat Rev Clin Oncol.* (2025) 22:163–81. 10.1038/s41571-024-00984-x 39820025

[59] Zemanek T Nova Z Nicodemou A. Tumor-Infiltrating lymphocytes and adoptive cell therapy: state of the art in colorectal, breast and Lung Cancer. *Physiol Res.* (2023) 72:S209–24. 10.33549/physiolres.935155 37888965 PMC10669950

[60] Keam SJ. Lifileucel: first approval. *Mol Diagn Ther.* (2024) 28:339–44. 10.1007/s40291-024-00708-y 38625642

[61] Lybaert L Lefever S Fant B Smits E De Geest B Breckpot K Challenges in neoantigen-directed therapeutics. *Cancer Cell.* (2023) 41:15–40. 10.1016/j.ccell.2022.10.013 36368320

[62] Ben-Avi R Farhi R Ben-Nun A Gorodner M Greenberg E Markel G Establishment of adoptive cell therapy with tumor infiltrating lymphocytes for non-small cell lung cancer patients. *Cancer Immunol Immunother.* (2018) 67:1221–30. 10.1007/s00262-018-2174-4 29845338 PMC11028292

[63] Mandracci G Soliman N El Khawanky N. Overcoming immune therapy resistance in Cancer through innate immune reprogramming. *Int J Mol Sci.* (2025) 26:9554. 10.3390/ijms26199554 41096817 PMC12525178

[64] Zahavi D Hodge JW. Targeting immunosuppressive adenosine signaling: a review of potential immunotherapy combination strategies. *Int J Mol Sci.* (2023) 24:8871. 10.3390/ijms24108871 37240219 PMC10218801

[65] Kandra P Nandigama R Eul B Huber M Kobold S Seeger W Utility and drawbacks of Chimeric Antigen Receptor T Cell (CAR-T) therapy in Lung Cancer. *Front Immunol.* (2022) 13:903562. 10.3389/fimmu.2022.903562 35720364 PMC9201083

[66] Zhao T You J Wang C Li B Liu Y Shao M Cell-based immunotherapies for solid tumors: advances, challenges, and future directions. *Front Oncol.* (2025) 15:1551583. 10.3389/fonc.2025.1551583 40356763 PMC12066282

[67] Tsai KK Komanduri KV. Tumor-Infiltrating lymphocyte therapy for the treatment of metastatic melanoma. *Am J Clin Dermatol.* (2025) 26:733–45. 10.1007/s40257-025-00957-5 40549109 PMC12436572

[68] Mohammad A Yurina A Simonyan T Chistyakov D Salman R Zornikova K Modular (universal) CAR-T platforms in vivo: a comprehensive systematic review. *Front Immunol.* (2024) 15:1409665. 10.3389/fimmu.2024.1409665 39712013 PMC11659234

[69] Zhu H Blum RH Bernareggi D Ask EH Wu Z Hoel HJ Metabolic reprograming via deletion of CISH in human iPSC-Derived NK cells promotes in vivo persistence and enhances anti-tumor activity. *Cell Stem Cell.* (2020) 27:224–37.e6. 10.1016/j.stem.2020.05.008. 32531207 PMC7415618

[70] ClinicalTrials.gov *NCT03215810: Nivolumab and Tumor-Infiltrating Lymphocytes (TIL) in Advanced Non-Small Cell Lung Cancer.* Bethesda, MD: U.S. National Library of Medicine (2017).

[71] National Cancer Institute *NCI-2017-01966: Nivolumab, Tumor-Infiltrating Lymphocytes, Chemotherapy, and Aldesleukin in Treating Patients With Recurrent or Stage IV Non-Small Cell Lung Cancer.* Bethesda, MD: National Institutes of Health (2017).

[72] ClinicalTrials.gov *IOV-LUN-202: A Phase II Study of LN-145 (Autologous Tumor-Infiltrating Lymphocytes) in Patients With Metastatic Non-Small Cell Lung Cancer.* Bethesda, MD: U.S. National Library of Medicine (2018).

[73] ClinicalTrials.gov *NCT05902520: Adoptive Cell Therapy Using Cancer Specific CD8+ Tumor Infiltrating Lymphocytes in Adult Patients With Solid Tumors.* Bethesda, MD: U.S. National Library of Medicine (2023).

[74] ClinicalTrials.gov *NCT06455917: TIL Therapy in Non-Small-Cell Lung Cancer (NSCLC) Patients (BaseTIL-02).* Bethesda, MD: U.S. National Library of Medicine (2024).

